# Sustained high-efficiency daily diafiltration using a cytokine-adsorbing membrane in the treatment of patients with severe sepsis

**DOI:** 10.1186/cc9535

**Published:** 2011-03-11

**Authors:** O Nishida, T Nakamura, N Kuriyama, Y Hara, K Moriyama, M Yumoto, Y Shimomura

**Affiliations:** 1 Fujita Health University School of Medicine, Toyoake, Japan

## Introduction

Sustained high-efficiency daily hemodiafiltration using a cytokine-adsorbing membrane (SHEDD-fA) is an effective modality for sepsis treatment. Here we describe the effectiveness of SHEDD-fA, which makes the best use of three principles for solute removal, in the treatment of severe sepsis.

## Methods

Twenty-nine septic shock patients were analyzed retrospectively. SHEDD-fA was initiated after adequate fluid resuscitation and catecholamine support. Operation conditions were QB = 150 ml/minute, QF = 1,500 ml/hour (post-dilution) and QD = 300 to 500 ml/minute using an HD machine over 8 to 12 hours daily. For the purpose of maximizing cytokine adsorption efficiency, we used a large-size (2.1 m^2^) PMMA dialyzer.

## Results

### Decrease in blood IL-6 level

**Figure 1:**
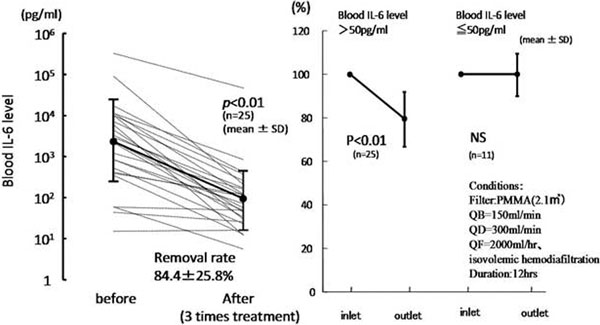
**Decrease in blood IL-6 level over 3 days (left) and the removal ratio in one pass (right)**.

SHEDD-fA was performed for 3 days. The percentage of IL-6 removed from the blood was 84.4 ± 25.8% (mean ± SD; *P *< 0.01; *n *= 25; Figure 1). In addition, we simultaneously assayed both inlet and outlet IL-6 and found a 21.0 ± 13.4% (*P *< 0.01; *n *= 25) removal ratio, showing that IL-6 is effectively removed after one pass through the hemofilter. Moreover, depressed monocytic HLA-DR ratio was improved from 40.6 to 51.9% in one typical case.

### Hemodynamics and PaO_2_/FiO_2 _improvement

In 22 out of the 29 septic shock patients, significant decreases in the catecholamine index/mean blood pressure were observed 3 hours after the initiation of SHEDD-fA (*P *< 0.01). In septic ARDS patients, PaO_2_/FiO_2 _was significantly improved at 1 hour (*P *< 0.01). The improvement of the abovementioned parameters continued afterwards for 72 hours. As a result, 13 of 16 patients survived.

## Conclusions

We propose the use of a large-size, cytokine-adsorbing hemofilter (PMMA or AN69 based membrane) and the selection of a suitable duration modality in the treatment of severe sepsis.

